# Thermoluminescence can be used to study lipid peroxidation in photosynthetic organisms

**DOI:** 10.1007/s11120-025-01171-4

**Published:** 2025-09-23

**Authors:** José M. Ortega

**Affiliations:** 1 Instituto de Bioquímica Vegetal y Fotosíntesis, cicCartuja, Universidad de Sevilla-CSIC, Seville, 41092 Spain; 2 Departamento de Bioquímica Vegetal y Biología Molecular, Facultad de Biología, Universidad de Sevilla, Seville, 41012 Spain

**Keywords:** Lipid peroxidation, Oxidative stress, Photosynthesis, Thermoluminescence

## Abstract

Oxidants attack lipids with carbon-carbon double bonds, causing the formation of lipid peroxyl radicals and hydroperoxides through a process called lipid peroxidation. Different aldehydes, including malondialdehyde, can also be formed as secondary products. The thiobarbituric acid reactive substances (TBARS) test is commonly used as an assay to measure lipid peroxidation, and its determination is based on spectrophotometric quantification of malondialdehyde. However, the TBARS test is not entirely specific for lipid peroxidation analysis because of the presence of other malondialdehyde sources and the possibility of reaction with other oxidation products. High temperature thermoluminescence technique is a useful method for studying lipid peroxidation in photosynthetic organisms. This technique measures the luminescence emission generated at high temperatures by some of the final products of lipid peroxidation. The breakdown of lipid peroxides is caused by high temperatures, which leads to the formation of carbonyl species in an excited triplet state. When chlorophyll molecules receive energy from excited carbonyls, they release this energy as luminescence once they settle into their ground state. Multiple studies have observed significant thermoluminescence emission bands at high temperatures caused by the energy transfer of lipid peroxidation by-products to chlorophyll. The band peaking at 115–130 °C correlates well with the concentration of different lipid peroxidation products. This band is an extremely sensitive in vivo indicator of the effects of stress conditions in photosynthetic materials. This technique has several benefits when used for lipid peroxidation assays. It is non-invasive, does not require the addition of external probes, and offers sensitive and continuous monitoring of peroxide levels.

## Lipid peroxidation

Participation of molecular oxygen in metabolic processes results in the formation of reactive oxygen species (ROS), including superoxide, hydrogen peroxide, hydroxyl radical, and singlet oxygen (Mittler 2002). These oxygen species break down various cellular components such as lipids, proteins, and nucleic acids, leading to the degradation of these substances and ultimately resulting in cell death and tissue damage. One of the primary processes caused by ROS, by direct or indirect pathways, is lipid peroxidation, which is particularly pronounced in membranes containing high levels of polyunsaturated fatty acids (PUFAs), such as those of chloroplast membranes (Gutteridge and Halliwell 1990). In photosynthetic organisms, a significant amount of oxygen is produced in thylakoids near powerful oxidation–reduction systems, which easily convert oxygen into hazardous superoxide and generate singlet oxygen that could trigger lipid peroxidation reactions (Gutteridge and Halliwell 1990).

Lipid peroxidation occurs through a process in which reactive oxidants, including ROS, target lipids with carbon-carbon double bonds, resulting in hydrogen abstraction from a carbon atom, oxygen insertion, and lipid peroxyl radical and hydroperoxide formation (Yin et al. 2011). The overall process comprises three steps: initiation, propagation, and termination (Girotti 1998; Yin et al. 2011). Prooxidants, such as the hydroxyl radical, abstract an allylic hydrogen to form the carbon-centered lipid radical (L•) in the initiation step. In the propagation phase, a lipid radical (L•) rapidly reacts with oxygen to form a lipid peroxy radical (LOO•), which subsequently captures a hydrogen atom from another lipid molecule, forming a fresh lipid radical (L•) and a lipid hydroperoxide (LOOH), sustaining a chain reaction. During the termination reaction, antioxidants like vitamin E donate a hydrogen atom to LOO• radicals, forming a corresponding vitamin E radical that subsequently reacts with another LOO• to create nonradical products. Once lipid peroxidation is initiated, chain reactions occur until termination products are produced.

Membrane lipids are ultimately destroyed by lipid peroxidation, resulting in the formation of multiple breakdown products, the primary being lipid hydroperoxides (Dianzani and Barrera 2008). A range of different aldehydes, including malondialdehyde (MDA), propanal, hexanal, and 4-hydroxynonenal (4-HNE), can be produced as secondary products (Esterbauer and Cheeseman 1990; Esterbauer et al. 1991; Kodali et al. 2020). MDA is the most mutagenic of these products, whereas 4-HNE has been found to be the most toxic (Esterbauer et al. 1990).

## Assay methods for lipid peroxidation

Lipid peroxidation is typically identified and measured by assessing for some of the secondary products of the process, such as MDA, 4-HNE, or other carbonyl compounds, or by directly examining the levels of the main primary products, lipid hydroperoxides (Heath and Parker 1968; Pryor and Castle 1984; Halliwell and Gutteridge 1984; Chatterjee and Agarwal 1988; Gutteridge and Halliwell 1990; Yagi et al. 1996; Kodali et al. 2020; Abeyrathne et al. 2021). The methods used to determine lipid hydroperoxides are simple and inexpensive, although their practical application in living organisms may be limited by the short lifetime of these molecules. Their sensitivities are lower than those of measurements of the secondary products (Yagi et al. 1996; Abeyrathne et al. 2021).

The thiobarbituric acid reactive substances (TBARS) assay is one of the most frequently employed tests to determine lipid oxidation. This method measures the concentration of MDA by reacting it with thiobarbituric acid to form a product that gives a pink colour, that can be measured using a visible or fluorescent spectrophotometer (Bird and Draper 1984; Zeb and Ullah 2016). TBARS assay is a simple, reproducible and low-cost method. This test does not exclusively identify lipid peroxidation, because there are other sources of MDA. The TBARS test lacks specificity for MDA due to its lability to detect a wide range of lipid breakdown products, namely thiobarbituric acid–reactive substances (Halliwell and Gutteridge 1984). Furthermore, the TBARS test is not ideally suited for measuring MDA in vivo because the oxidation products of sucrose, ascorbate, and deoxyribose can react with TBARS to form adducts that show a similar absorbance spectrum to MDA. In addition, MDA is readily metabolized, resulting in a very short half-life in vivo (Halliwell and Gutteridge 1984).

Alternative chromatographic techniques have been developed for the analysis of MDA and other secondary or primary oxidation products. High-Performance liquid chromatography (HPLC) coupled with visible (HPLC-UV/Vis) or fluorescence (HPLC-FL) detection, and gas chromatography, equipped with mass spectrometry, are extremely sensitive and discriminatory methods to measure lipid peroxidation, yet they require significantly more time than TBARS assay, and the equipment cost is much higher (Yeo et al. 1994; Kodali et al. 2020; Abeyrathne et al. 2021; Chen et al. 2022).

Often, lipid peroxidation secondary products as MDA and 4-HNE, cause protein modifications that can be analysed and quantified by Western blotting and immunoassays methodologies (Spickett 2013; Kodali et al. 2020; Lee et al. 2022; Zarkovic et al. 2024). Enzyme-linked immunosorbent assay (ELISA) technique for identifying MDA and 4-HNE protein adducts have resulted in the development of advanced methodology for detecting lipid peroxidation (Duryee et al. 2010; Weber et al. 2013; Zarkovic et al. 2024). The routine use of these chemical methods for lipid oxidation assessment in microorganisms is impractical because of the substantial amount of biological material required for analysis, which can be time-consuming and unsuitable for high-throughput testing.

Since certain lipid peroxidation products like ^1^O_2_ and excited carbonyls emit light, oxidative stress can be tracked in vivo using chemiluminescence techniques (Sies 1987). Low-intensity photon emission has become a commonly employed technique in the field of animal sciences to track oxidative stress (Sato et al. 1992; Devaraj et al. 1997; Albertini and Abuja 1998). Several studies have shown that this luminescence signal is related to other indicators of lipid peroxidation (Albertini and Abuja 1998; Doi et al. 2002; Guajardo et al. 2002), thereby validating the idea that low-level chemiluminescence can be used to measure lipid peroxidative damage. Chemiluminescence methods provide sensitive, fast and simple assays for quantitative determination of the hydroperoxides, and have a higher sensibility compared to the other absorption techniques (Kodali et la. 2020).

Electron paramagnetic (EPR) and nuclear magnetic (NMR) resonance spectroscopies have been extensively used to measured both primary and secondary products of lipid peroxidation. EPR spin trapping is an analytical technique for the detection and analysis of free radicals produced during lipid oxidation, that are short-lived and can rarely be detected directly (Cui et al. 2017). NMR spectroscopy is a very useful technology to study lipid peroxidation since it can investigate the changes in the starting materials as well as the evolution of the oxidation products during the process (Alexandri et al. 2017). Both technologies, however, require very expensive equipment.

The use of fluorescent dyes/probes in confocal microscopy methodology is a powerful tool for visualizing lipid peroxidation in living cells. These combined techniques allow for the detection of lipid hydroperoxides with high sensitivity and selectivity, and provides very valuable information about the possible role of peroxidation in cellular pathology (Barden and Decker 2017; Abeyrathne et al. 2021).

A technique based on thermoluminescence has been proposed as a helpful approach for identifying lipid peroxidation in photosynthetic organisms (Vavilin and Ducruet 1998; Havaux 2003; Ducruet and Vass 2009). Gradual heating of chlorophyll-containing materials to temperatures exceeding 70 °C frequently results in an increase in chlorophyll luminescence emission. The enhanced luminescence intensity observed at high temperatures was termed high temperature thermoluminescence or HTL (Matorin et al. 1989; Venediktov et al. 1989). Thermal decomposition of peroxidised lipids leads to the formation of carbonyls in a triplet state and, subsequently, the migration of excitation energy towards chlorophylls, which then release it as luminescence when they return to their ground state (Cadenas 1984; Venediktov et al. 1989; Vavilin et al. 1991; Hideg and Vass 1993; Sharov et al. 1996; Vavilin and Ducruet 1998).

In the thermal decomposition of peroxidised lipids, a wide HTL band centred at around 130 °C (HTL2 band) is produced. The intensity of the HTL2 band correlates well with the concentration of different lipid peroxidation products as determined by conventional methods (Vavilin et al. 1991; Stallaert et al. 1995; Vavilin and Ducruet 1998; Ducruet and Vavilin 1999). Studies have shown that the HTL2 peak´s intensity is linked to the amount of MDA present in algae, leaves, and thylakoids that have undergone oxidative stress (Kafarov et al. 1988; Venediktov et al. 1989; Vavilin et al. 1991, 1998; Merzlyak et al. 1992). The intensity of this band showed a correlation with the amount of TBARS present in tobacco leaves treated with a fungal elicitor (Stallaert et al. 1995).

The HTL technology offers discriminatory and sensitive tracking of peroxide concentration, potentially resolving some problems associated with traditional lipid peroxidation detection techniques. It is a simple, inexpensive and non-invasive method; it does not require the addition of external probes, and offers very sensitive and continuous monitoring of peroxide levels. In addition, this technique requires the use of small amounts of biological material. In future research, HTL technique could potentially be used as a rapid screening method in photosynthetic organisms before to the use of more complex or time-consuming techniques (Repetto et al. 2015).

## Photosynthetic thermoluminescence

Light emission from previously irradiated solid-state materials occurs when they are warmed up in the dark at specific temperatures (Chen and Kirsh 1981; Vass and Govinjee 1996; Vass 2003). This phenomenon, called thermoluminescence (TL), can be observed in numerous natural minerals and artificially created solid states (including semiconductors, organic solids, and metal-organic compounds), as well as in complex biological systems such as the photosynthetic apparatus. In solids, exposed electrons and holes can become separated and trapped in energy wells, where the probability of recombination via radiative or non-radiative processes is very low. When the temperature is increased, the thermal energy may become sufficient to allow electrons and holes to escape and recombine from the traps. When this happens through radiative processes, the sample will start emitting light and continue emitting light as the temperature rises until the traps are emptied.

In 1957, Arnold and Sherwood first recorded the observation of TL in green plant material. Previously, Strehler and Arnold (1951), based on their observation of delayed luminescence from chloroplasts, proposed that some absorbed light energy is stored in the photosynthetic apparatus in long-lived, highly stable trap states. This light emission, termed as delayed light emission (DLE), originates from chlorophyll fluorescence in photosystem II (PSII), resulting from the recombination of previously light-separated charge pairs (Amesz and van Gorkom 1978; Sane and Rutherford 1986; Vass and Inoue 1992). Charge pairs separated photochemically stabilize on PSII due to activation energy barriers that prevent charge recombination. Charge recombination occurs via several pathways, one of which leads to the regeneration of an exciton in the chlorophyll antenna at a relatively low efficiency with a likelihood of deactivation as fluorescence (Rappaport et al. 2005).

The precursor charge pairs generated by light can stabilize at low temperatures; subsequent heating in the dark leads to DLE, which is commonly known as thermoluminescence (TL) or thermally stimulated delayed luminescence (Arnold and Sherwood 1957; Tollin and Calvin 1957). The TL technique involves recording the emission of luminescence as a sample is warmed after exposure to irradiation at a sufficiently low temperature to minimize the recombination rate of charge pairs being studied (Vass and Inoue 1992; Inoue 1996; Vass and Govindjee 1996; Ducruet 2003; Ducruet and Vass 2009; Sane et al. 2012).

The TL technique enables the identification of various types of charge pairs in PSII through successive emission bands upon progressive warming. A B band of TL, peaking around 25 °C, is observed following a sequence of short flashes in both isolated photosynthetic membranes and intact systems. The emission is caused by the recombination of S_2_/S_3_Q_B_^−^ pairs (Demeter et al. 1982; Inoue and Shibata 1982; Rutherford et al. 1982, 1984a, b; Demeter and Vass 1984). Treatment with a PSII-inhibiting herbicide, such as diuron or atrazine, which blocks the electron transfer from Q_A_ to Q_B_, results in the formation of a Q band peaking at around 5 °C, caused by the S_2_Q_A_^−^ recombination (Demeter et al. 1982; Demeter and Vass 1984). A C band at approximately 55 °C can also be detected in particular conditions. This TL band is caused by D^+^Q_A_^−^ recombination, where D^+^ is the oxidized form of tyrosine D on the inactive branch of PSII (Desai et al. 1975; Demeter et al. 1993; Johnson et al. 1994).

A particular type of luminescence emission, referred to as “afterglow” (AG), is typically achieved in intact photosynthetic materials after irradiation with far-red light (Hideg et al. 1991; Miranda and Ducruet 1995). AG emission is associated with the fraction of PSII centers in the S_2_/S_3_Q_B_ nonradiative state immediately following pre-illumination, where the arrival of an electron transferred from the stroma via the cyclic or chlororespiratory pathway(s) results in the S_2_/S_3_Q_B_^−^ radiative state, which emits luminescence. The emission can be optimally recorded with a sharp TL band peaking at about 45 °C by a linear temperature gradient (Sundblad et al. 1988; Hideg et al. 1991; Miranda and Ducruet 1995; Vavilin and Ducruet 1998; Roncel and Ortega 2005).

## High temperature thermoluminescence in photosyntetic organisms

In addition to the reversal of electron transfer reactions in photosynthesis, other processes result in light emission when chlorophyll-containing samples are heated. Several groups have reported high-temperature thermoluminescence bands above 60 °C, which are not due to charge recombination of PSII redox components but likely result from an energy transfer of lipid peroxidation products toward chlorophyll (Venediktov et al. 1989; Vavilin et al. 1991, 1998; Hideg and Vass 1993; Vavilin and Ducruet 1998).

Arnold and Sherwood (1957) initially documented a high-temperature TL emission peaking at 125 °C in chlorophyll found in irradiated dried thylakoids. Chlorophyll-containing materials experience a boost in luminescence emission when heated to temperatures exceeding 70 °C without prior illumination (Arnold and Sherwood 1957; Matorin et al. 1989; Hideg and Vass 1993; Vavilin and Ducruet 1998). This increased luminescence intensity at high temperatures is referred to as high-temperature thermoluminescence or HTL (Matorin et al. 1989; Venediktov et al. 1989). This type of TL is emitted within the same 650–750 nm spectral range as chlorophyll fluorescence and PSII luminescence, but it occurs independently of prior illumination. HTL emission has no connection to photosynthesis, with the exception that dark-excited chlorophylls act as luminescence emitters in the red spectrum (Ducruet and Vavilin 1999).

Two distinct types of HTL bands, with maxima temperatures at 75 °C/90 °C (Hideg and Vass 1993; Stallaert et al. 1995; Marder et al. 1998) or 115 °C/140 °C (Venediktov et al. 1989; Vavilin et al. 1991; Merzlyak et al. 1994; Merzlyak and Hendry 1994; Vavilin and Ducruet 1998; Vavilin et al. 1998), have been identified after oxidative stress treatments in photosynthetic materials. A HTL band at approximately 73 °C, described by Desai et al. (1982), is attributed to chemiluminescence from chlorophyll resulting from membrane destruction. A band with a maximum temperature of 75 °C, caused by oxidative treatments in isolated spinach chloroplasts, was also described by Hideg and Vass (1993). It was considered to result from a temperature-enhanced interaction between molecular oxygen and the photosynthetic membrane, possibly involving membrane lipid peroxidation during exposure to high temperatures (Hideg and Vass 1993). A similar band was discovered in tobacco leaves treated with a fungal elicitor (Stallaert et al. 1995) and in greening barley leaves (Marder et al. 1998). The band displayed the highest temperature of 90 °C when heated at a rate of 30 °C per minute and a lowest temperature of 70 °C when heated at a rate of 3 °C per minute (Stallaert et al. 1995).

Around 1990, researchers at the University of Moscow observed HTL bands above 60 °C, with a primary band at 130 °C, in algae or leaves exposed to oxidative stress (Kafarov et al. 1988; Venediktov et al. 1989; Vavilin et al. 1991; Merzlyak et al. 1992). Vavilin and Ducruet (1998) showed that treatments causing oxidative damage to membrane lipids produced three HTL bands: a small 60–75 °C band (HTL1 band), a significant rise in the 115–130 °C peaks (HTL2 band), and another band above 150 °C (HTL3 band). It is clear from the work of these authors that the type of emitted band is contingent on the water content of the sample. The HTL1 band was exclusively observed under wet conditions. To convert this band to an HTL2 band, dehydration of the sample prior to measurement and a dry analysis environment are necessary. The HTL2 band was visible when the sample was exposed to a dry atmosphere in a standard TL setup, with liquid nitrogen functioning as a drying agent in a sealed environment. In contrast, upon heating samples (chloroplast, algae, leaf squares) in a water medium to 100 °C to prevent desiccation, as typically done in TL photosynthetic studies, only a band became apparent at approximately 75 °C. The nature of the HTL3 band, with spans 151–158 °C, remains unclear. Nitrogen inhibited the band, that remained stable throughout the high-light treatment of thylakoids, a period during which lipid peroxidation took place. Consequently, this band seems unrelated to the thermolysis of lipid peroxidation products and could instead be caused by the heat-induced autoxidation of leaf compounds (Vavilin and Ducruet 1998).

A distinct thermoluminescence band peaking at approximately 65 °C, even without initial light exposure, has been identified in specific *Arabidopsis thaliana* and *Phaseolus vulgaris* biotypes (Ducruet 2013). This band is inhibited by freezing temperatures below the negative nucleation point or by replacing air with nitrogen gas in the TL cell, suggesting both oxidative mechanisms and compartmentalization. This thermo-chemiluminescence emission can be regarded as an HTL1 emission and possibly results from the radiative oxidation of certain compounds found in *Arabidopsis thaliana* and *Phaseolus vulgaris* (Vavilin and Ducruet 1998; Ducruet 2013; Ducruet, personal communication).

## Origin of the HTL2 band

Polyunsaturated fatty acids undergo chain oxidation reactions, resulting in the formation of cycloperoxides (Gardner 1989). Vavilin and Ducruet (1998) proposed that the HTL2 band originates from the thermal decomposition of lipid cyclic peroxides, as it remained unchanged after treatment with quenchers of active oxygen species, scavengers of free radicals, or replacing oxygen with argon. The HTL2 band observed in an uncovered leaf sample did not decrease after flushing the compartment with nitrogen, indicating that peroxides did not form during warming, but disappeared when the sample remained wet, suggesting that lipid peroxides were hydrolyzed before radiative thermolysis occurred (Ducruet and Vavilin 1999). Consequently, the HTL1 band recorded in wet conditions is a pseudo-band resulting from the competition between radiative thermolysis and heat-enhanced non-radiative hydrolysis (Ducruet and Vavilin 1999; Hideg and Vass 1993; Marder et al. 1998; Vavilin and Ducruet 1998).

The breakdown of lipid cycloperoxides stimulated by high temperatures leads to the formation of carbonyl compounds, such as MDA, in the triplet state excited (Halliwell and Gutteridge 1985; Vavilin and Ducruet 1998). Excited carbonyl molecules quickly transfer energy to chlorophyll molecules, which then release it as luminescence when they return to their ground state (Matorin et al. 1989; Venediktov et al. 1989; Vavilin and Ducruet 1998; Vavilin et al. 2002). Cilento and co-authors demonstrated that energy from triplet carbonyls is efficiently transferred to chlorophyll (Campa and Cilento 1984; Nassi and Cilento 1984). The observation that tyrosine, an efficient quencher of triplet species (Rivas-Suarez et al. 1983), led to a considerable decrease in the intensity of the HTL2 band (Vavilin and Ducruet 1998), indicating the involvement of triplet carbonyls in TL emission. The appearance of the HTL2 band following the interaction between LOO• and (or) LO• radicals can be ruled out because numerous external free-radical scavengers were unable to decrease the HTL intensity in preilluminated thylakoids (Vavilin and Ducruet 1998). These authors also suggested that the hydroperoxides breakdown cannot explain HTL2 emission.

## Instruments for high temperature thermoluminescence

All HTL reported measurements were performed using devices produced in the same laboratories. Although there is a company (Photon Systems Instruments PSI, Czech Republic) which produces a commercial TL instrument that can be used for HTL measurements. The home-made devices largely share the same basic principles (Vass et al. 1981; Vavilin et al. 1991; Ducruet and Miranda 1992; Vavilin and Ducruet 1998; Ducruet and Vavilin 1999; Roncel et al. 2016). Essentially, a setup should have a sample holder that can be cooled and heated at a controlled temperature, a temperature sensor capable of accurately measuring the sample’s temperature, and a red-sensitive photomultiplier tube, preferably an end-window type, along with a high-voltage power supply.

A custom-built device developed by Dr. Jean-Marc Ducruet for the Institute of Plant Biochemistry and Photosynthesis (University of Seville and CSIC) to measure thermoluminescence within the ranges of standard (0–85 °C) and high-temperature (20–160 °C) is described here. Further information about these systems can be found in the references cited (Repetto et al. 2015; Roncel et al. 2016; Castell et al. 2021, 2022; Díaz-Santos et al. 2025).

A PC computer controlled temperature regulation and signal recording through a National Instrument DAQ-Pad1200 interface using dedicated software. The sample cuvette comprised a 2 cm diameter horizontal chamber and a base copper film. A Thermocoax electrically insulated resistor heater (France) was powered by a variable (0 to 5 A) computer-controlled power supply and positioned below the chamber for temperature control. A homemade power amplifier controlled the temperature by supplying a variable current (or voltage) to the resistor heater, which was rated at a maximum of 40 V and 5 A. A thermocouple situated between the filter and the heating element was used to regulate the temperature. Luminescence emission was detected using a Hamamatsu H5701-50 photomultiplier module from Japan. The luminescence was filtered through a high-pass Schott RG 645 filter, which has the maximum transmission for wavelengths greater than 670 nm, and then focused onto the photomultiplier via a light guide to prevent the photomultiplier windows from heating during high-temperature liquid measurements. Data acquisition, signal analysis, and graphical simulation were performed as previously described (Ducruet and Miranda 1992; Zurita et al. 2005; Ducruet et al. 2011).

Cell suspensions of algae or cyanobacteria were filtered for HTL measurement using a 0.22-mm cellulose ester membrane or a piece of 0.45 mm Whatman filter paper. Then, the dehydrated cell suspension was placed on the sample cuvette and pressed with a metal washer to ensure thermal contact with the resistor heater plate. Leaf disc samples were placed into the sample cuvette by either pressing a metal grid onto the leaf or sticking the leaf disc onto a piece of aluminum or copper adhesive sheet commonly used in electronics. This can prevent wilting or popping up of leaf samples due to dryness above 100 °C. The sample was flushed with N_2_ gas for 15 min before and during the experiment to desiccate the samples and prevent oxidation caused by high temperatures. The samples were incubated in the dark for 10 min at 20 °C, and the luminescence emission was subsequently recorded as the samples were heated from 10 °C to 160 °C at a rate of 0.1 °C per second. During TL warming, caution should be exercised to prevent the sample from being covered by a window and to allow it to dry.

## HTL2 band as a sensitive indicator of plant stress

The HTL technique has been employed to investigate in vivo lipid peroxidation in photosynthetic organisms subjected to diverse abiotic stress conditions. The defensive properties of the violaxanthin cycle were studied in a mutant of *Arabidopsis thaliana*, npq1, which lacks functional violaxanthin deepoxidase (Havaux and Niyogi 1999). HTL-detected lipid peroxidation was more pronounced in npq1 than in its wild-type counterpart. Chilling stress significantly increased lipid peroxidation, ultimately causing photooxidative damage which led to leaf bleaching and tissue necrosis in npq1. The results show that the violaxanthin cycle safeguards thylakoid membrane lipids against photooxidation specifically.

The phototolerance of three chlP transgenic tobacco lines, which were deficient in tocopherols (vitamin E), was estimated using HTL and fluorescence measurements (Havaux et al. 2003). Exposure to high light and low temperature caused a swift reduction in PSII activity, which was subsequently followed by lipid peroxidation after a delay of roughly 4 h. The phenomenon became more intense in leaves deficient in tocopherol. The results indicated that tocopherols protect thylakoid membranes from photo-destruction by lipid peroxidation.

The photosynthetic anomalies in the atrazine-resistant soybean cell line STR7, which has a single S268P mutation in the chloroplastic psbA gene that codes for the PSII’s D1 protein, have been investigated using different spectroscopic methods (Roncel et al. 2007). The analysis of the HTL2 band demonstrated significantly increased lipid peroxidation in STR7 cells compared to WT cells. The authors suggested that the mutation in STR7 line results in a slower transfer of electrons from Q_A_ to Q_B_ in the PSII complex, thereby increasing the likelihood of singlet oxygen formation, which may trigger lipid peroxidation in thylakoid membranes.

The emissions of HTL were studied in plants of the Ljgln2-2 photorespiratory mutant from *Lotus japonicus* that were grown either under photorespiratory or non-photorespiratory conditions (García-Calderón et al. 2019). Plants of both WT and Ljgln2-2 mutant varieties grown under non-photorespiratory conditions exhibited a significantly higher intensity of the HTL2 band compared to plants grown under photorespiratory conditions. These findings showed increased peroxidation of chloroplast lipids when photorespiration was inhibited and implied that photorespiration may have a protective effect on chloroplasts against lipid peroxidation.

Vavilin and Ducruet (1998) have reported that treatments involving low temperature and intense light that induce oxidative damage to cell membrane lipids, resulting in the appearance of a small HTL1 band and a substantial increase in the intensity of a HTL2 band. Leaves of *Acer platanoides* and *Aesculus hippocastanum* desiccate significantly in the dark, showed a substantial rise in chlorophyll chemiluminescence peaks at 120 °C, which is associated with the accumulation of lipid peroxidation products in chloroplast membranes (Merzlyak et al. 1992).

The photosynthetic response of algae to nutrient deficiency was also examined with the help of HTL technique. High lipid peroxidation levels were observed in *Phaeodactylum tricornutum* cells grown in iron-deficient conditions under low light intensity (Roncel et al. 2016). These results indicate that iron deficiency may trigger the occurrence of photoinhibitory processes on the acceptor side and subsequently lead to the production of singlet oxygen at a very low light intensity, which are typically not photoinhibitory. Vavilin et al. (2002) found a rise in HTL when *Chlorella* (*C*.) *pyrenoidosa* was cultivated under conditions where nitrogen or phosphorus was in short supply, and the light levels were moderate. The HTL emission remained low when *C. pyrenoidosa* or other species were cultivated in nutrient-rich media, even when they were exposed to intense light. Therefore, nutrient availability could be crucial for cell resistance against light-induced membrane lipid peroxidation (Vavilin et al. 2002).

The HTL technique has also been employed to assess the impacts of certain biotic stress in plants. Infection of *Nicotiana benthamiana* with the pepper mild mottle virus resulted in a substantial rise in the HTL2 band (Sajnani et al. 2007). Viral infection-induced changes in cell metabolism, like impaired Calvin cycle function, may contribute to oxidative stress development. Stallaert et al. (1995) found that treating tobacco leaves with the fungal elicitor cryptogein led to a significant rise in the intensity of the HTL band. These results indicate that cryptogein triggers the formation of reactive species within the chloroplast, likely participating in the lipid peroxidation process.

Measurements of HTL are beneficial for studying the significance of lipid peroxidation within natural phytoplankton communities (Vavilin et al. 2002). Selective detection of HTL in the red part of the spectra corresponding to the maximum chlorophyll emission (670–680 nm) allows for a clear distinction between the luminescence produced by chlorophyll-containing photosynthetic organisms and the chemiluminescence emitted by chlorophyll-free particulate matter, which typically appears as blue-shifted compared to chlorophyll luminescence. Vavilin et al. (2002) have reported that the intensity of lipid peroxidation reactions in phytoplankton was influenced by daily fluctuations in light intensity. But the comparison of the daily dynamics of the HTL in phytoplankton collected at varied locations indicated that light intensity is not the sole factor contributing to the rise in lipid peroxides content within the cells.

The HTL technique has also been applied to the toxicological assessment of certain chemicals using photosynthetic species. The effects of six environmental compounds on *Chorella vulgaris* cells were investigated using this technique: bromobenzene and diethanolamine as chemical intermediates, propyl gallate as an antioxidant, indium nitrate as a semiconductor, sodium monofluoroacetate as a pesticide, and chloroquine as an antimalarial drug (Repetto et al. 2015). The analysis of the HTL2 band revealed a high degree of lipid peroxidation in the photosynthetic membranes of *Chlorella* following incubation with four tested chemicals: bromobenzene, sodium monofluoroacetate, diethanolamine, and chloroquine. Detection of a rise in thermoluminescence at 120–130 °C occurred when cultures of the unicellular green alga *C. pyrenoidosa* were exposed to 10 µM of the oxidative stress promoter methyl viologen for 5 h (Vavilin and Ducruet 1998). Additionally, Hörcsik et al. (2007) found that photodestruction of D1 protein in *C. pyrenoidosa* caused by Cr (VI) resulted in enhanced oxidative stress and lipid peroxidation, as evidenced by the appearance of an HTL2 band.

## HTL bands in cianobacteria

Only a handful of studies have employed the HTL technique to investigate lipid peroxidation in cyanobacteria. Incubation of *Synechocystis* sp. PCC 6803 cells with methyl viologen, a herbicide that promotes the oxidation of membrane lipids, resulted in a considerable increase in the amplitude of the HTL2 band, and also triggered thermoluminescence at approximately 60–75 °C (Vavilin et al. 2002). The HTL technique has also been applied to study the impacts of mutations on the cytochrome b559 in PSII function in the thermophilic cyanobacteria *Thermosynechococcus elongatus* (Guerrero et al. 2014). Cells with an αR18S mutation showed a high level of lipid peroxidation when cultured under normal conditions without photoinhibition. This mutation could modify the interaction between cytochrome b559 and Q_B_ or the plastoquinone pool, leading to a high concentration of Q_B_^−^, which may increase the likelihood of ^1^O_2_ formation, a highly reactive and toxic species. This may trigger the peroxidation of unsaturated lipids within photosynthetic membranes.

## Data Availability

No datasets were generated or analysed during the current study.

## References

[CR1] Abeyrathne EDNS, Nam K, Ahn DU (2021) Analytical methods for lipid oxidation and antioxidant capacity in food systems. Antioxidants 10:1587 34679722 10.3390/antiox10101587 PMC8533275

[CR2] Albertini R, Abuja PM (1998) Monitoring of low-density lipoprotein oxidation by low-level chemiluminescence. Free Radic Res 29:75–83 9733024 10.1080/10715769800300091

[CR3] Alexandri E, Ahmed R, Siddiqui H, Choudhary MI, Tsiafoulis CG, Gerothanassis IP (2017) High resolution NMR spectroscopy as a structural and analytical tool for unsaturated lipids in solution. Molecules 22:1663 28981459 10.3390/molecules22101663 PMC6151582

[CR5] Amesz J, van Gorkom HJ (1978) Delayed fluorescence in photosynthesis. Ann Rev Plant Physiol 29:47–66

[CR4] Arnold WA, Sherwood HK (1957) Are chloroplasts semiconductors? Proc Natl Acad Sci USA 43:105–114 16589982 10.1073/pnas.43.1.105 PMC528393

[CR6] Bird RP, Draper HH (1984) Comparative studies on different methods of malonaldehyde determination. In: Packer L (ed) Methods in enzymology, vol 105. Academic Press, London, pp 299–305 10.1016/s0076-6879(84)05038-2 6727668

[CR7] Cadenas E (1984) Biological chemiluminescence. Photochem Photobiol 40:823–830 6395146 10.1111/j.1751-1097.1984.tb04657.x

[CR8] Campa A, Cilento G (1984) Low-level luminescence from microsomes exposed to enzymatic systems that generate triplet species. Arch Biochem Biophys 235:673–678 6517606 10.1016/0003-9861(84)90243-1

[CR9] Castell C, Bernal-Bayard P, Ortega JM, Roncel M, Hervás M, Navarro JA (2021) The heterologous expression of a plastocyanin in the diatom phaeodactylum tricornutum improves cell growth under iron-deficient conditions. Physiol Plant 171:277–290 33247466 10.1111/ppl.13290

[CR10] Castell C, Díaz-Santos E, Heredia-Martínez LG, López-Maury L, Ortega JM, Navarro JA, Roncel M, Hervás M (2022) Iron deficiency promotes the lack of photosynthetic cytochrome c550 and affects the binding of the luminal extrinsic subunits to photosystem II in the diatom *Phaeodactylum tricornutum*. Int J Mol Sci 23:12138 36292994 10.3390/ijms232012138 PMC9603157

[CR11] Chatterjee SN, Agarwal S (1988) Liposomes as membrane model for study of lipid peroxidation. Free Rad Biol Med 4:51–72 2830175 10.1016/0891-5849(88)90011-1

[CR12] Chen R, Kirsh Y (1981) In: Pamplin B (ed) Analysis of thermally stimulated processes. Pergamon, Oxford

[CR13] Chen Z, Wu X, Chiba H, Hui S-P (2022) Investigating oxidized lipids in an omics way: oxidative lipidomics in biological applications using mass spectrometry. Med Mass Spectrom 6:72–84

[CR14] Cui L, Lahti PM, Decker EA (2017) Evaluating electron paramagnetic resonance (EPR) to measure lipid oxidation lag phase for shelf—life determination of oils. J Am Oil Chem Soc 94:89–97

[CR16] Demeter S, Vass I (1984) Charge accumulation and recombination in photosystem II studied by thermoluminescence. I. Participation of the primary acceptor Q and secondary acceptor B in the generation of thermoluminescence of chloroplasts. Biochim Biophys Acta – Bioenerg 764:24–32

[CR15] Demeter S, Droppa M, Vass I, Horvath G (1982) Thermoluminescence of chloroplasts in the presence of photosystem II herbicides. Photobiochem Photobiophys 4:163–168

[CR17] Demeter S, Goussias C, Bernat G, Kovacs L, Petrouleas V (1993) Participation of the g = 1.9 and g = 1.82 EPR forms of the semiquinone iron complex, Q_A_^–^Fe^2+^ of photosystem II in the generation of the Q and C thermoluminescence bands, respectively. FEBS Lett 336:352–356 8262261 10.1016/0014-5793(93)80836-j

[CR18] Desai TS, Sane PV, Tatake VG (1975) Thermoluminescence studies on spinach leaves and Euglena. Photochem Photobiol 21:345–350 1208661 10.1111/j.1751-1097.1975.tb06682.x

[CR19] Desai TS, Tatake VG, Sane PV (1982) High temperature peak on glow curve of photosynthetic membrane. Photosynth 16:129–133

[CR20] Devaraj B, Usa M, Inaba H (1997) Biophotons: ultraweak light emission from living systems. Curr Opin Solid State Mat Sci 2:188–193

[CR21] Dianzani M, Barrera G (2008) Pathology and physiology of lipid peroxidation and its carbonyl products. In: Álvarez S, Evelson P (eds) Free radical pathophysiology. Transworld Research Network, Kerala, India, pp 19–38

[CR22] Díaz-Santos E, Heredia-Martínez LG, López-Maury L, Hervás M, Ortega JM, Navarro JA, Roncel M (2025) Combined effect of temperature and different light regimes on the photosynthetic activity and lipid accumulation in the diatom *Phaeodactylum tricornutum*. Plants 14:329 39942891 10.3390/plants14030329 PMC11820123

[CR23] Doi H, Iwasaki H, Masubuchi Y, Nishigaki R, Horie T (2002) Chemiluminescence associated with the oxidative metabolism of salicylic acid in rat liver microsomes. Chem Biol Interact 140:109–119 12076519 10.1016/s0009-2797(02)00004-2

[CR24] Ducruet JM (2003) Chlorophyll thermoluminescence of leaf discs: simple instruments and progress in signal interpretation open the way to new ecophysiological indicators. J Exp Bot 54:2419–2430 14565948 10.1093/jxb/erg268

[CR25] Ducruet JM (2013) Pitfalls, artefacts and open questions in chlorophyll thermoluminescence of leaves or algal cells. Photosynth Res 115:89–99 23720191 10.1007/s11120-013-9859-5

[CR26] Ducruet JM, Miranda T (1992) Graphical and numerical analysis of the thermoluminescence and fluorescence Fo emission in photosynthetic material. Photosynth Re 33:15–27 10.1007/BF00032979 24408444

[CR28] Ducruet JM, Vass I (2009) Thermoluminescence: experimental. Photosynth Res 101:195–204 19551489 10.1007/s11120-009-9436-0

[CR29] Ducruet JM, Vavilin D (1999) Chlorophyll high-temperature thermoluminescence emission as an indicator of oxidative stress. Perturbating effects of oxygen and leaf water content. Free Radic Res 31:187–192 10.1080/10715769900301491 10694058

[CR27] Ducruet JM, Serrano A, Roncel M, Ortega JM (2011) Peculiar properties of chlorophyll thermoluminescence emission of autotrophically or mixotrophically grown *Chlamydomonas reinhardtii*. J Photochem Photobiol B 104:301–307. 10.1016/j.jphotobiol.2011.02.014 21402481 10.1016/j.jphotobiol.2011.02.014

[CR30] Duryee MJ, Klassen LW, Schaffert CS, Tuma DJ, Hunter CD, Garvinc RP, Andersonc DR, Thiele GM (2010) Malondialdehyde–acetaldehyde adduct is the dominant epitope after MDA modification of proteins in atherosclerosis. Free Rad Biol Med 49:1480–1486 20696236 10.1016/j.freeradbiomed.2010.08.001 PMC2952714

[CR31] Esterbauer H, Cheeseman KH (1990) Determination of aldehydic lipid peroxidation products: malonaldehyde and 4-hydroxynonenal. Methods Enzymol 186:407–421 2233308 10.1016/0076-6879(90)86134-h

[CR32] Esterbauer H, Eckl P, Ortner A (1990) Possible mutagens derived from lipids and lipid precursors. Mut Res 238:223–233 2342513 10.1016/0165-1110(90)90014-3

[CR33] Esterbauer H, Schaur RJ, Zollner Z (1991) Chemistry and biochemistry of 4-hydroxynonenal, malonaldehyde and related aldehydes. Free Rad Biol Med 11:81–128 1937131 10.1016/0891-5849(91)90192-6

[CR34] García-Calderón M, Betti M, Márquez AJ, Ortega JM, Roncel M (2019) The afterglow thermoluminescence band as an indicator of changes in the photorespiratory metabolism in the model legume *Lotus japonicas*. Physiol Plan 166:240–250 10.1111/ppl.12916 30628087

[CR35] Gardner HW (1989) Oxygen radical chemistry of polyunsaturated fatty acids. Free Radical Biol Med 7:65–86 2666279 10.1016/0891-5849(89)90102-0

[CR36] Girotti AW (1998) Lipid hydroperoxide generation, turnover, and effector action in biological systems. J Lipid Res 39:1529–1542 9717713

[CR38] Guajardo MH, Terrasa AM, Catalá A (2002) Retinal fatty acid binding protein reduce lipid peroxidation stimulated by long-chain fatty acid hydroperoxides on rod outer segments. Biochim Biophys Acta 1581:65–74 12020634 10.1016/s1388-1981(02)00121-x

[CR37] Guerrero F, Zurita JL, Roncel M, Kirilovsky D, Ortega JM (2014) The role of the high potential form of the cytochrome *b*559: Study of *Thermosynechococcus elongatus* mutants. Biochim Biophys Acta-Bioenerg 837:908–919 10.1016/j.bbabio.2014.02.024 24613347

[CR39] Gutteridge JMC, Halliwell B (1990) The measurement and mechanism of lipid peroxidation in biological systems. Trends Biochem Sci 15:129–135 2187293 10.1016/0968-0004(90)90206-q

[CR40] Halliwell B, Gutteridge JMC (1984) Oxygen toxicity, oxygen radicals, transition metals and disease. Biochem J 219:1–14 6326753 10.1042/bj2190001 PMC1153442

[CR41] Halliwell B, Gutteridge JMC (1985) Free radical in biology and medicine Vol 2. Clarendon Press, Oxford

[CR42] Havaux M (2003) Spontaneous and thermoinduced photon emission: new methods to detect and quantify oxidative stress in plants. Trends Plant Sci 8:409–413 13678906 10.1016/S1360-1385(03)00185-7

[CR44] Havaux M, Niyogi KK (1999) The Violaxanthin cycle protects plants from photo-oxidative damage by more than one mechanism. Proc Nat Acad Sci USA 96:8762–8767 10411949 10.1073/pnas.96.15.8762 PMC17590

[CR43] Havaux M, Lütz C, Grimmet B (2003) Chloroplast membrane photostability in ChlP Transgenic tobacco plants deficient in tocopherols. Plant Physiol 132:300–310 12746535 10.1104/pp.102.017178 PMC166975

[CR45] Heath RL, Parker L (1968) Photoperoxidation in isolated chloroplasts. I. Kinetics and stoichiometry of fatty acid peroxidation. Arch Biochem Biophys 125:189–198 5655425 10.1016/0003-9861(68)90654-1

[CR47] Hideg E, Vass I (1993) The 75ºC thermoluminescence band of green tissues: chemiluminescence from membrane-chlorophyll interaction. Photochem Photobiol 58:280–283

[CR46] Hideg E, Kobayashi M, Inaba H (1991) The Far red induced slow component of delayed light from chloroplasts is emitted from photosystem II. Evidence from emission spectroscopy. Photosynth Res 29:107–112 24415112 10.1007/BF00035381

[CR48] Hörcsik ZT, Kovács L, Láposi R, Mészáros I, Lakatos G, Garab G (2007) Effect of chromium on photosystem 2 in the unicellular green alga, chlorella pyrenoidosa. Photosynth 45:65–69

[CR49] Inoue Y (1996) Photosynthetic thermoluminescence as a simple probe of photosystem II electron transport. In: Amesz J, Hoff AJ (eds) Biophysical techniques in photosynthesis. Kluwer Academic, Dordrecht, pp 93–107

[CR50] Inoue Y, Shibata K (1982) Thermoluminescence from photosynthetic apparatus. In: Govindjee (ed) Photosynthesis: energy conversion by plants and bacteria, vol 1. Academic, New York, pp 507–533

[CR51] Johnson GN, Boussac A, Rutherford AW (1994) The origin of 40–50ºC thermoluminescence bands in photosystem II. Biochim Biophys Acta – Bioenerg 1184:85–92

[CR52] Kafarov RS, Shenderova LV, Matorin DN, Venedictov PS (1988) Fiziolog Rastenii 35:458–463

[CR53] Kodali ST, Kauffman P, Kotha SR, Yenigalla A, Veeraraghavan R, Pannu RS, Hund TJ, Satoskar AR, McDaniel JC, Maddipati RK, Parinandi NL (2020) Oxidative lipidomics: analysis of oxidized lipids and lipid peroxidation in biological systems with relevance to health and disease. In: Berliner J, Parinandi NL (eds) Measuring oxidants and oxidative stress in biological systems. Springer Nature Switzerland, pp 61–92 33411442

[CR54] Lee Y-J, Linc Y-C, Liaof C-C, Changg Y-S, Huangi Y-H, Tsaij I-J, Chenk J-H, Ling S-H, Linm Y-F, Hsiehi T-W, Chenc Y-S, Wuc C-Y, Changh C-C, Lini C-Y, (2022) Using anti-malondialdehyde-modified peptide adduct autoantibodies in serum of Taiwanese women to diagnose primary Sjogren’s syndrome. Clin Biochem 108:27–41 35843269 10.1016/j.clinbiochem.2022.07.002

[CR55] Marder JB, Droppa M, Caspi V, Raskin VI, Horvath G (1998) Light-independent thermoluminescence from greening barley leaves: evidence for involvement of oxygen radicals and free chlorophylls. Physiol Plant 104:713–719

[CR56] Matorin DN, Vavilin DV, Kafarov RS, Venediktov PS (1989) High temperature thermoluminescence of chlorophyll as a method for study of lipid peroxidation in plants. Dokl Akad Nauk SSSR 309:764–768

[CR59] Merzlya MN, Hendry GAF, Atherton NM, Zhigalova TV, Pavlov VK, Zhitenva OV (1994) Pigment destruction, lipid peroxidation and free radical formation in leaves during autumn senescence. Biohhimiya 58:240–249

[CR57] Merzlyak MN, Hendry GAF (1994) Free radical metabolism, pigment degradation and lipid peroxidation in leaves during senescence. Proc R Soc Edin-Biol Sci 102:459–471

[CR58] Merzlyak MN, Pavlov VK, Zhigalova TV (1992) Effect of desiccation on chlorophyll high temperature chemiluminescence in Acer platanoides L. and aesculus Hippocastanum L. leaves. J Plant Physiol 139:629–631

[CR60] Miranda T, Ducruet JM (1995) Characterization of the chlorophyll thermoluminescence afterglow in dark-adapted or far-red illuminated plant leaves. Plant Physiol Biochem 33:689–699

[CR61] Mittler R (2002) Oxidative stress, antioxidants and stress tolerance. Trends Plant Sci 7:405–410 12234732 10.1016/s1360-1385(02)02312-9

[CR62] Nassi L, Cilento G (1984) Excitation of micelle-solubilized chlorophyll during the peroxidase-catalyzed aerobic oxidation of isonicotinic acid hydrazide. Arch Biochem Biophy 229:220–225 10.1016/0003-9861(84)90147-4 6703694

[CR63] Pryor WA, Castle L (1984) Chemical methods for the detection of lipid hydroperoxides. Methods Enzymol 105:293–305 6727667 10.1016/s0076-6879(84)05037-0

[CR64] Rappaport F, Cuni A, Xiong L, Sayre R, Lavergne J (2005) Charge recombination and thermoluminescence in photosystem II. Biophys J 88:1948–1958 15653722 10.1529/biophysj.104.050237 PMC1305247

[CR65] Repetto G, Zurita JL, Roncel M, Ortega JM (2015) Thermoluminescence as a complementary technique for the toxicological evaluation of chemicals in photosynthetic organisms. Aquat Toxicol 158:88–97 25461748 10.1016/j.aquatox.2014.11.002

[CR66] Rivas-Suarez E, Catalani LH, Bechara EJH, Cilento G (1983) Quenching of chemically and enzymatically generated triplet acetone by tyrosine and 3,s-dihalogenderivatives. Photochem Photobio 37:93–97

[CR67] Roncel M, Ortega JM (2005) Afterglow thermoluminescence band as a possible early indicator of changes in the photosynthetic electron transport in leaves. Photosynth Res 84:167–172 16049770 10.1007/s11120-004-7311-6

[CR68] Roncel M, Yruela I, Kirilovsky D, Guerrero F, Alfonso M, Picorel R, Ortega JM (2007) Changes in photosynthetic electron transfer and state transitions in an herbicide-resistant D1 mutant from soybean cell cultures. Biochim Biophys Acta 1767:694–702 17442261 10.1016/j.bbabio.2007.02.017

[CR69] Roncel M, González-Rodríguez AA, Naranjo B, Bernal-Bayard P, Lindahl M, Hervás M, Navarro JA, Ortega JA (2016) Iron deficiency induces a partial Inhibition of the photosynthetic electron transport and a high sensitivity to light in the diatom phaeodactylum tricornutum. Front Plant Sci 7:1050 27536301 10.3389/fpls.2016.01050 PMC4971056

[CR70] Rutherford AW, Crofts AR, Inoue Y (1982) Thermoluminescence as a probe of photosystem II photochemistry. The origin of the flash induced glow peaks. Biochim Biophys Acta – Bioenerg 682:457–465

[CR71] Rutherford AW, Renger G, Koike H, Inoue Y (1984a) Thermoluminescence as a probe of photosystem II. The redox and protonation States of the secondary acceptor Quinone and the O*2*-evolving enzyme. Biochim Biophys Acta – Bioenerg 767:548–556

[CR72] Rutherford AW, Zimmermann JL, Mathis P (1984b) The effect of herbicides on components of the PSII reaction centre measured by EPR. FEBS Lett 165:156–162

[CR73] Sajnani C, Zurita JL, Roncel M, Ortega JM, Barón M, Ducruet JM (2007) Changes in photosynthetic metabolism induced by tobamovirus infection in Nicotiana benthamiana studied in vivo by thermoluminescence. New Phytol 175:120–130. 17547672 10.1111/j.1469-8137.2007.02074.x

[CR74] Sane PV, Rutherford AW (1986) In: Fork J DC (ed) Thermoluminescence from photosynthetic membranes. In: light emission by plants and Bacteria, Amesz. Academic, New York, pp 329–360

[CR75] Sane PV, Ivanov AG, Öquist G, Hüner NPA (2012) Thermoluminescence. In: Eaton-Rye JJ, Tripathy BC, Sharkey TD (eds) Photosynthesis: plastid Biology, energy conversion and carbon Assimilation; advances in photosynthesis and respiration. Springer, Dordrecht, The Netherlands; Vol. 34, Chapter 19, pp 445–474

[CR76] Sato T, Miyazawa T, Kobayashi M, Furukawa H, Inaba H (1992) Low-level chemiluminescence and life span of drosophila melanogaster. Geront 38:50–58 10.1159/000213306 1612461

[CR77] Sharov VS, Briviba K, Sies H (1996) Assessment of the C-525 laser dye as a chemiluminescence sensitizer for lipid peroxidation in biological membranes: a comparison with chlorophyll-a. Free Radical Biol Med 21:833–843 8902529 10.1016/0891-5849(96)00236-5

[CR78] Sies H (1987) Intact organ spectrophotometry and single-photon counting. Arch Toxicol 60:138–143 3304203 10.1007/BF00296967

[CR79] Spickett CM (2013) The lipid peroxidation product 4-hydroxy-2-nonenal: advances in chemistry and analysis. Redox Biol 1:145–152 24024147 10.1016/j.redox.2013.01.007 PMC3757682

[CR80] Stallaert VM, Ducruet JM, Tavernier E, Blein JP (1995) Lipid peroxidation in tobacco leaves treated with the elicitor cryptogein: evaluation by high-temperature thermoluminescence emission and chlorophyll fluorescence. Biochim Biophys Acta 1229:290–295

[CR81] Strehler B, Arnold W (1951) Light production by green plants. J Gen Physiol 34:809–820 14850702 10.1085/jgp.34.6.809 PMC2147280

[CR82] Sundblad LG, Schröder WP, Akerlund H-E (1988) S-state distribution and redox state of QA in barley in relation to luminescence decay kinetics. Biochim Biophys Acta 973:47–52

[CR83] Tollin G, Calvin M (1957) The luminescence of chlorophyll-containing plant material. Proc Natl Acad Sci USA 43:895–908 16590106 10.1073/pnas.43.10.895 PMC528548

[CR84] Vass I (2003) The history of photosynthetic thermoluminescence. Photosynth Res 76:303–318 16228589 10.1023/A:1024989519054

[CR86] Vass I, Govindjee (1996) Thermoluminescence from the photosynthetic apparatus. Photosynth Res 48:117–126 24271292 10.1007/BF00041002

[CR87] Vass I, Inoue Y (1992) Thermoluminescence in the study of photosystem II. The photosystems: function und molecular Biology, Barber j. Elsevier, Amsterdam, pp 259–294

[CR85] Vass I, Horvath G, Herczeg T, Demeter S (1981) Photosynthetic energy conservation investigated by thermoluminescence. Activation energies and half-lives of thermoluminescence bands of chloroplasts determined by mathematical resolution of glow curves. Biochim Biophys Acta 634:140–152 7470496 10.1016/0005-2728(81)90134-1

[CR88] Vavilin DV, Ducruet JM (1998) The origin of 120–130°C thermoluminescence bands in chlorophyll-containing material. Photochem Photobiol 68:191–198

[CR89] Vavilin DV, Matorin DN, Kafarov RS, Bautina AL, Venediktov PS (1991) High-temperature thermoluminescence of chlorophyll in lipid peroxidation. Biol Membr 8:89–98

[CR90] Vavilin DV, Ducruet JM, Matorin DN, Venediktov PS, Rubin AB (1998) Membrane lipid peroxidation, cell viability and photosystem II activity in the green alga *Chlorella pyrenoidosa* subjected to various stress conditions. J Photochem Photobiol B 42:233–239

[CR91] Vavilin DV, Matorin DN, Rubin AB (2002) High-temperature thermoluminescence of chlorophyll as a method to study lipid peroxidation in planktonic algae. Arch Hydrobiol 153:685–701

[CR92] Venediktov PS, Matorin DN, Kafarov RS (1989) Chemiluminescence of chlorophyll upon lipid photoperoxidation in thylakoid membranes. Biofizika (Moscow) 34:241–245

[CR93] Weber D, Milkovic L, Bennett SJ, Griffiths HR, Zarkovic N, Grune T (2013) Measurement of HNE-protein adducts in human plasma and serum by ELISA. Comparison of two primary antibodies. Red Biol 1:226–233 10.1016/j.redox.2013.01.012 PMC3757688 24024156

[CR94] Yagi K, Komura S, Kojima H, Sun Q, Nagata N, Ohishi N, Nishikimi M (1996) Expression of human hydroperoxide glutathione peroxidase gene for protection of host cells from lipid hydroperoxide-mediated injury. Biochem Biophys Res Com 219:486–491 8605014 10.1006/bbrc.1996.0260

[CR95] Yeo HC, Helbock HJ, Chyu DW, Ames BN (1994) Assay of malondialdehyde in biological fluids by gas chromatography-mass spectrometry. Anal Biochem 220:391–396 7978283 10.1006/abio.1994.1355

[CR96] Yin H, Xu L, Porter NA (2011) Free radical lipid peroxidation: mechanisms and analysis. Chem Rev 111:5944–5972 21861450 10.1021/cr200084z

[CR97] Zarkovic Z, Gegotek A, Łuczaj W, Jaganjac M, Sunjic SB, Zarkovic K, Skrzydlewska E (2024) Overview of the lipid peroxidation measurements in patients by the enzyme-linked immunosorbent specific assay for the 4-hydroxynonenal-protein adducts (4-HNE-ELISA). Front Biosci (Landmark Ed) 29:153 38682198 10.31083/j.fbl2904153

[CR98] Zeb A, Ullah FA (2016) Simple spectrophotometric method for the determination of thiobarbituric acid reactive substances in fried fast foods. J. Anal. Methods Chem. 9412767 10.1155/2016/9412767 PMC4830699 27123360

[CR99] Zurita JL, Roncel M, Aguilar M, Ortega JM (2005) A thermoluminescence study of photosystem II back electron transfer reactions in rice leaves. Effects of salt stress. Photosynth Res 84:131–137 16049765 10.1007/s11120-004-6427-z

